# Clinically early-stage *CSPα* mutation carrier exhibits remarkable terminal stage neuronal pathology with minimal evidence of synaptic loss

**DOI:** 10.1186/s40478-015-0256-5

**Published:** 2015-11-26

**Authors:** Bruno A. Benitez, Nigel J. Cairns, Robert E. Schmidt, John C. Morris, Joanne B. Norton, Carlos Cruchaga, Mark S. Sands

**Affiliations:** Department of Medicine, Washington University School of Medicine, St. Louis, MO 63110 USA; Department of Neurology, Washington University School of Medicine, St. Louis, MO 63110 USA; Department of Pathology & Immunology, Washington University School of Medicine, St. Louis, MO 63110 USA; Department of Psychiatry, Washington University School of Medicine, St. Louis, MO 63110 USA; Department of Genetics, Washington University School of Medicine, St. Louis, MO 63110 USA; Hope Center for Neurological Disorders, Washington University School of Medicine, St. Louis, MO 63110 USA

**Keywords:** Neuronal ceroid lipofuscinosis, Cysteine string protein alpha, Synaptic loss, Neurodegenerative disease, Lysosome DNAJC5

## Abstract

Autosomal dominant adult-onset neuronal ceroid lipofuscinosis (AD-ANCL) is a multisystem disease caused by mutations in the *DNAJC5* gene. *DNAJC5* encodes Cysteine String Protein-alpha (*CSPα*), a putative synaptic protein. AD-ANCL has been traditionally considered a lysosomal storage disease based on the intracellular accumulation of ceroid material. Here, we report for the first time the pathological findings of a patient in a clinically early stage of disease, which exhibits the typical neuronal intracellular ceroid accumulation and incipient neuroinflammation but no signs of brain atrophy, neurodegeneration or massive synaptic loss. Interestingly, we found minimal or no apparent reductions in *CSPα* or synaptophysin in the neuropil. In contrast, brain homogenates from terminal AD-ANCL patients exhibit significant reductions in SNARE-complex forming presynaptic protein levels, including a significant reduction in *CSPα* and *SNAP-25*. Frozen samples for the biochemical analyses of synaptic proteins were not available for the early stage AD-ANLC patient. These results suggest that the degeneration seen in the patients with AD-ANCL reported here might be a consequence of both the early effects of *CSPα* mutations at the cellular soma, most likely lysosome function, and subsequent neuronal loss and synaptic dysfunction.

## Background

The neuronal ceroid lipofuscinoses (NCLs) are the most common group of inherited neurodegenerative diseases in children [1]. NCLs are a genetically, clinically and pathologically heterogeneous group of disorders, unified by the presence of intracellular accumulation of autofluorescent ceroid-lipofuscin aggregates (a complex mixture of proteins, lipids and metals) [1, 2]. The age at onset of clinical symptoms and differences in ultrastructural features of the storage material underlie the nosological spectrum of NCLs [1, 2]. NCLs are traditionally considered lysosomal storage diseases (LSD), even though several of the mutant proteins are not primary lysosomal proteins. The function of some of these proteins is largely unknown and their relationship to lysosome function is unclear [3].

Adult-onset NCLs (ANCL) represent up to 10 % of NCL cases [4]. ANCL patients exhibit the typical autofluorescent granular osmiophilic deposits (GROD) in neurons and skin cells [4, 5]. In recent years, the progress in sequencing technology has supported the identification of a plethora of novel disease-causing genes in multiple families with ANCL-like clinical features and GROD-type storage material. This indicates that the genetic architecture of ANCL is more varied and complex than previously thought [3].

Most NCLs have a recessive pattern of inheritance; one exception is the autosomal dominant, adult onset neuronal ceroid lipofuscinosis (AD-ANCL) (MIM #162350). AD-ANCL patients are asymptomatic until around the third decade [5]. The first symptom is usually new-onset seizures (tonic-clonic type) followed by rapid cognitive decline, motor impairment and finally, death in the absence of visual impairments [5]. The primary clinical and pathological features of AD-ANCL include progressive and widespread neurodegenerative processes resulting in remarkable cortical and cerebellar atrophy [5]. Nearly all of our current understanding of AD-ANCL is based on analysis of postmortem tissue from terminal cases. To our knowledge, there is no report characterizing both early and late pathology events of AD-ANCL cases.

AD-ANCL is caused by a heterozygous point mutation (p.L115R) and an in-frame codon deletion (p.L116Δ) in the DnaJ (Hsp40) homolog, subfamily C, member 5 (*DNAJC5)* gene [6–8]. The *DNAJC5* gene encodes *CSPα*. *CSPα* is a cytosolic and membrane-bound co-chaperone with a putative neuroprotective function [9]. The function of *CSPα* in the central nervous system is primarily deduced from data collected from CSPα-deficient mice and flies, which exhibit massive age-dependent neurodegeneration affecting mainly active neurons and photoreceptors [9, 10]. *CSPα* is widely considered a member of the presynaptic machinery responsible for proteostasis and synapse maintenance [11–13]. Defective synaptic soluble NSF attachment protein receptor (SNARE)-complex assembly, due mostly but not exclusively to the reduction in soluble NSF attachment protein-25 (*SNAP-25*) levels, appears to be responsible for the neurodegeneration seen in CSPα-deficient mice [10, 11, 14, 15]. There are approximately 20 additional proteins that demonstrate altered expression patterns in the absence of *CSPα* in mice [13] that can also be part of its pathogenic cascade.

The histological hallmark of AD-ANCL is the intracellular accumulation of autofluorescent storage material (AFSM) [4, 5]. Although there are similarities in the neurodegenerative process between AD-ANCL patients and CSPα-deficient mice, no ceroid accumulation has been reported in CSPα-deficient mice or flies [12, 16]. Currently, it is unknown how mutations in *DNAJC5/CSPα* result in the formation of AFSM. A dominant negative effect was initially proposed as the pathogenic mechanism for AD-ANCL-causing mutations in *CSPα* based on both its inheritance pattern and a reduction in neuropil* CSPα* staining in terminal AD-ANCL cases [7, 12, 17].

In this report, we describe the clinical and pathological features of a patient in a clinically early stage of the disease with all the pathological cellular changes found in terminal AD-ANCL cases but with no apparent reduction in *CSPα* or synaptophysin in the neuropil. In contrast, terminal AD-ANCL patients exhibit significant reductions of presynaptic protein levels, including a significant reduction of *CSPα*.

## Materials and methods

### Mutation analysis

*DNAJC5*-Exon4 (Forward, 5'-ggaaggcagtatccccacctggaac-3'; Reverse, 5'-cggcacagtgtcagtgccctcc-3') was amplified on Applied Biosystems (Applied Biosystems, Carlsbad, California, USA) 96-Well GeneAmp® PCR System 9700 Thermal Cyclers using a touchdown protocol. PCR products were amplified under the same conditions (25-μl volume containing 10× PfuUltra™ HF reaction buffer (Stratagene, La Jolla, California, USA), 5× Betaine (Sigma-aldrich, St Louis, USA), 100 μM each dNTP, 200 nM each primer, 0.4 PfuUltra™ High-Fidelity DNA Polymerase (Promega); PCR profile: 94 °C followed by 34 cycles of 45 s at 94 °C, 45 s at 62°, and 1 min at 72 °C). PCR product purification was completed with Exosap-IT (USB Corporation). Sequencing was performed both in forward and reverse direction using BigDye® Terminator v3.1 Cycle Sequencing Kit (ABI) on an ABI 3130 sequencer. Sequence traces were analyzed using Sequencher (v4.7, Gene Codes Corp, Ann Arbor, Michigan, USA).

### Neuropathological Methods

After death, the consent for brain removal was obtained from next-of-kin. Briefly, the left hemibrain was preserved in 10 % phosphate-buffered formalin for neuropathologic assessment; the right hemibrain was snap frozen and stored at −80 °C for biochemical studies [18]. Unfortunately, no flash frozen tissue was obtained from the early stage AD-ANCL patient. Tissue blocks were taken from the frontal, temporal, parietal, and occipital lobes, amygdala, hippocampus, basal ganglia, thalamus, brainstem, cerebellum, and spinal cord when available. Following formic acid (95 %) pretreatment for 5 min, histology was undertaken and included hematoxylin and eosin and a modified Bielschowsky silver impregnation. Immunohistochemistry was performed using the following primary antibodies: glial fibrillary acidic protein (*GFAP*, 1:2,000; Dako, Glostrup, Denmark), ionized calcium-binding adapter molecule 1 (*Iba1*, 1:2,000; Wako Chemicals USA Inc., Richmond, VA), synaptophysin (1:1,000; Abcam, Cambridge, MA) and *CSPα/DNAJC5* (AB1576, 1:1,000, Millipore, Temecula, CA).

### Densitometry

Densitometry of synaptophysin and *CSPα/DNAJC5* immunoreactivity was assessed in the cortical ribbon of the middle frontal gyrus, an area available in all mutation carriers and non-carriers, and included the gyral crest and sulcal depth. The intensity of immunoreactivity (luminance measured as arbitrary values) was determined following immunohistochemistry of all cases treated in a single batch using Densita (MicroBrightField, Inc., Williston, VT) software.

### Ultrastructural methods

Small pieces of tissues from the right palm of formalin-fixed tissue were processed for transmission electron microscopy as previously described [5]. Briefly, specimens were fixed overnight in 3 % glutaraldehyde in Sorensen’s buffer, osmicated, dehydrated in graded alcohols and embedded in Epon. Thin sections were cut on an ultramicrotome, collected on mesh grids, stained with lead citrate and uranyl acetate and examined with a JEOL 1200 electron microscope.

### Immunoblotting

Cells were lysed in radioimmune precipitation assay (RIPA) buffer (50 mM Tris–HCl, pH 7.4, 150 mM NaCl, 1 % Nonidet P-40 and 0.25 % sodium deoxycholate) plus 1X phenylmethanesulfonylfluoride (PMSF) and 1X complete protease inhibitor mixture (Sigma, St. Louis, MO) for 10 min on ice and then spun at 14,000 rpm for 10 min at 4 °C. Protein was subjected to electrophoresis and transferred to PDVF membrane (BIO-RAD, Hercules, CA). The primary antibodies were diluted as follows: CSPα (ADI-VAP-SV003-E; Enzo life science) 1:5000, HSC70/HSP73 (ADI-SPA-816; Enzo life science) 1:1000, Synaptobrevin/VAMP2 (104211, Synaptic System) 1:10000, Syntaxin-1 (110011, Synaptic System) 1:10000, SNAP-25 ( SMI81, Covance) 1:10000, Synaptophysin (ab8049, abcam) 1:5000, Flotillin (C-7, sc-133153, Santa Cruz Biotechnology) 1:5000, β-Actin (A1978, Sigma-Aldrich) 1:5000. The membranes were then incubated with secondary antibodies, horseradish peroxidase-conjugated anti-mouse or anti-rabbit IgG (KPL, Gaithersburg, MD) diluted 1:2000 in 4 % nonfat dry milk PBS-T for 1 h at room temperature. Signals were visualized using Lumigen ECL Ultra (TMA-6) (Lumigen, Southfield, MI). Densitometric semi-quantification was performed using ImageJ software (National Institutes of Health).

### Analysis of Clinical Samples

**Fig. 1:**
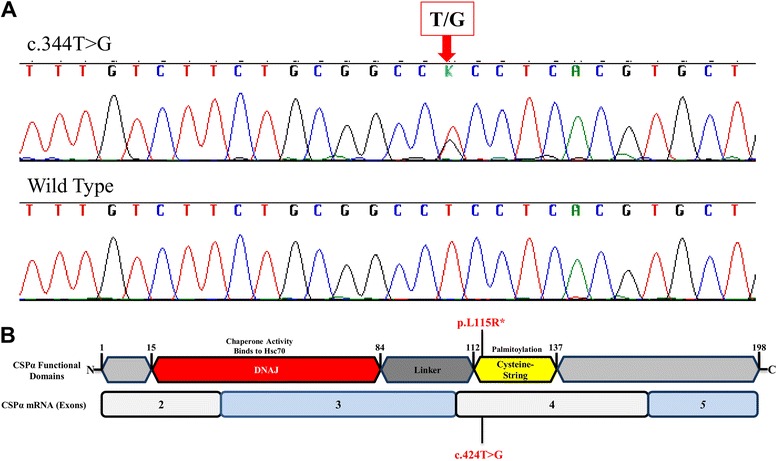
**a** DNA sequence traces of *DNAJC5* Exon 4 of an early stage AD-ANCL patient *(top)* and wild-type sequence from an age-gender matched control individual (*bottom*). **b** Schematic representation of the *DNAJC5* gene encoded protein (*top*) and main transcript (*bottom*). Mutation p.L115R is highlighted in *red*.

The Institutional Review Board (IRB) at the Washington University in Saint Louis School of Medicine approved the study. Prior to their participation, written informed consent was reviewed and obtained from family members. The Human Research Protection Office (HRPO) approval number for our ADRC Genetics Core family studies is 201104178. The neuropathological findings of AD-ANCL patients were published previously by Josephson et al. [5], a brief description is found in Table 1. *DNAJC5* mutation identification and screening was published by Benitez et al. [6]. The clinically early-stage patient with AD-ANCL belongs to the 7th generation of this family, identified as (7:3) in the pedigree (Fig. 1) [6].

**Table 1:** Demographics of AD-ANCL cases and healthy controls

Individual ID	Age at death	Gender	State	Brain wt. (g)	PMI (h)	Cause of death
1	37	F	*Case*	1160	4.5	Motor vehicle accident
2	49	F	*Case*	1010	13.5	Bronchopneumonia
3	50	F	*Case*	1050	17	Bronchopneumonia
4	50	M	*Case*	1110	22	Inanition
5	53	F	*Case*	1005	8	Parry’s disease
6	70	F	*Control*	1180	16	Carcinoma of colon
7	72	M	*Control*	980	5	Respiratory failure
8	72	F	*Control*	1060	16	Inanition
9	73	F	*Control*	1230	8	Carcinoma of lung
10	81	F	*Control*	1200	21	Myocardial infarction
11	89	F	*Control*	1100	33	Respiratory failure
12	90	F	*Control*	1050	22	Respiratory failure
13	90	M	*Control*	1320	6.5	Bronchopneumonia
14	91	M	*Control*	1170	8.5	Myocardial infarction
15	93	F	*Control*	1155	5	Respiratory failure

*Wt* weight, *PMI* post-mortem interval

### Statistical analyses

All data are shown as means ± SEM. For comparison of two groups, Student’s unpaired two-tailed t test was used. Data were analyzed using GraphPad Prism, version 5.00 (San Diego, CA).

## Case Presentation

### Mutation analyses of *DNAJC5*/*CSP*α/*NCL4B*

Sequencing analysis of exon 4 of the *DNAJC5* gene revealed a coding variant heterozygous transversion change at position c.344 (c.344 T > G) (Fig. 1a) which results in a leucine-to-arginine amino acid substitution (Fig. 1b). This mutation was previously found in other members of the same family with terminal AD-ANCL [6, 7].

### Clinical features of an early stage AD-ANCL patient

A member of a multigenerational family with AD-ANCL was evaluated as a research volunteer at age 30 and 33 years at the Knight Alzheimer's Disease Research Center, Washington University, St. Louis, Missouri, as part of the follow-up of family members. At both visits the patient was cognitively normal, had no neurological symptoms, and had an unremarkable neurological examination. Patient’s next evaluation was at 36 y, when patient reported experiencing multicolored visual hallucinations in the prior 12 months. More recently the patient reported apparent myoclonus affecting the upper extremities and had experienced several non-injurious falls. Three weeks prior to the assessment, patient had an accident while driving her automobile. The patient had no reported seizure episodes. The neurologic examination remained unremarkable and patient again was rated as cognitively normal. An awake electroencephalogram, however, showed bilateral epileptiform discharges. The patient was evaluated seven months later and began medication.

### Neuropathology of an early stage AD-ANCL patient

**Fig. 2:**
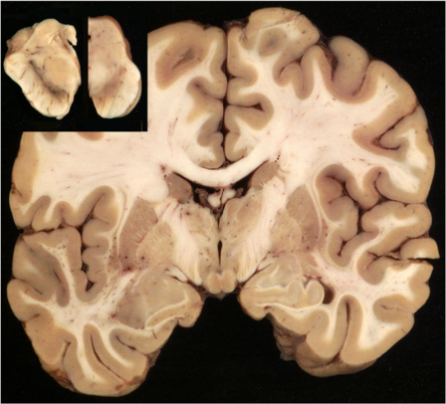
Macroscopic findings in the brain of an early stage AD-ANCL patient with *DNAJC5* p.L115R mutation. The cortical ribbon, basal ganglia, and medial temporal lobe structures at the level of the mammillary bodies are unremarkable. There is some depigmentation of the substantia nigra (Fig. 2, inset, *right panel*) in comparison with a normal subject (Fig. 2, inset, *left panel*)

**Fig. 3:**
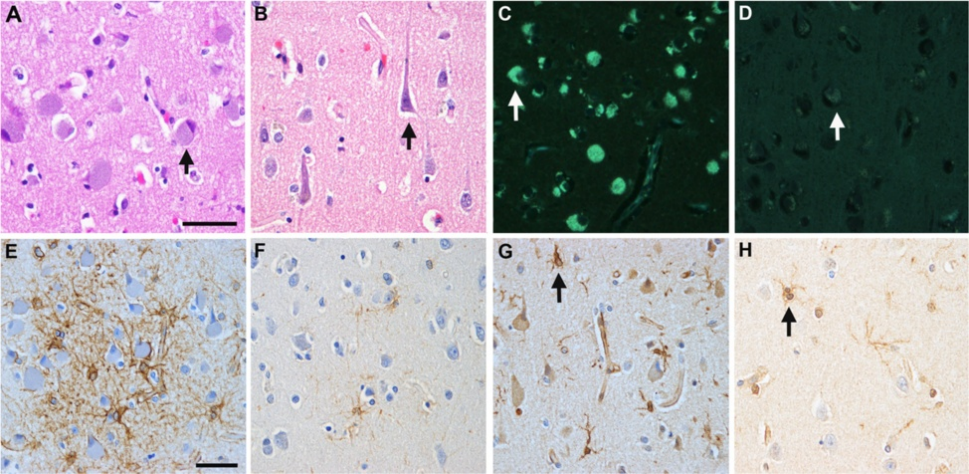
Microscopy of AD-ANCL patient with *DNAJC5* p.L115R mutation. **a** An enlarged cortical pyramidal neuron with eccentric nucleus in a mutation (MC) (*arrow*). **b** A typical pyramidal neuron in a non-mutation carrier (NC) control (*arrow*). Stain: Hematoxylin and eosin. **c** An enlarged neuron contains stored lipopigment which is autofluorescent under ultraviolet light (*arrow*). **d** NC control exhibits modest neuronal accumulation of lipofuscin (*arrow*). **e** *,* In a MC, there is patchy reactive astrocytosis (*GFAP* immunohistochemistry) **f** Sparse reactive astrocytes in a NC control. **g** There is a modest increase in the number of activated microglial cells in the frontal neocortex (*arrow*) in comparison with a NC control (*arrow*) (**h**) (**g** and **h**, *arrows* indicate microglial cells) (*Iba1* immunohistochemistry). **a**-**d** (scale bar in **a**) bar = 50 μm; (**e**-**h**) (scale bar in **e**) bar = 50 μm

Neuropathological examination of coronal slices revealed no apparent atrophy of the cortical and subcortical gray and white matter (Fig. 2). There were no changes found in the limbic system or the cerebellum of this patient. There was some depigmentation of the substantia nigra (Fig. 2, inset, right panel), compared to normal control (inset, left panel). We performed histological analysis of the brain and found a marked enlargement of the cortical pyramidal neurons (Fig. 3a). In affected neurons, there is displacement of the nucleus to the base of the apical dendrite and a markedly swollen cell body in comparison to the typical pyramidal shape of neurons in neocortical layer III (Fig. 3b). Neurons contained typical AFSM (Fig. 3c). We also found focal astrogliosis and relatively mild microgliosis adjacent to some swollen neurons in the frontal lobe neocortex (Fig. 3e and g, respectively).

**Fig. 4:**
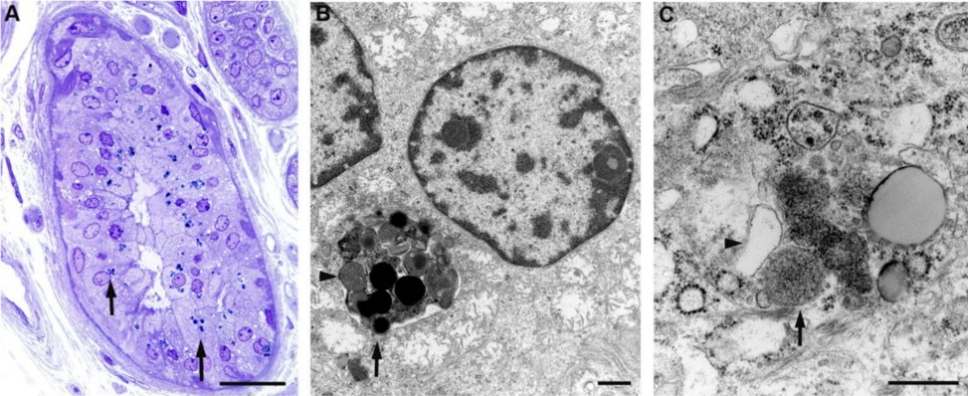
Fine structure of an early stage AD-ANCL patient with *DNAJC5* p.L115R mutation. **a** Palmar sweat gland with cuboidal secretory cells containing lipofuscin granules (*arrows*). **b** A cell containing dense and granular osmiophilic lipopigment (*arrow*). **c** Granular lipopigment (*arrow*) and a lipid globule (*arrowhead*) within a cytosomal membrane. **a** Toluidine blue; (**b** and **c**), Epon-embedded, uranyly acetate stain. Bars: (**a**), 100 μm; (**b**), 1 μm; (**c**), 500 nm

We also found cuboidal secretory cells containing lipofuscin granules in a palmar sweat gland (Fig. 4a). Electron microscopy revealed cells containing dense and granular osmiophilic lipopigment (Fig. 4b) as well as granular lipopigment and a lipid globule within a cytosomal membrane (Fig. 4c).

### No changes in levels of *CSPα* or synaptophysin in the brains of an early stage AD-ANCL patient

**Fig. 5:**
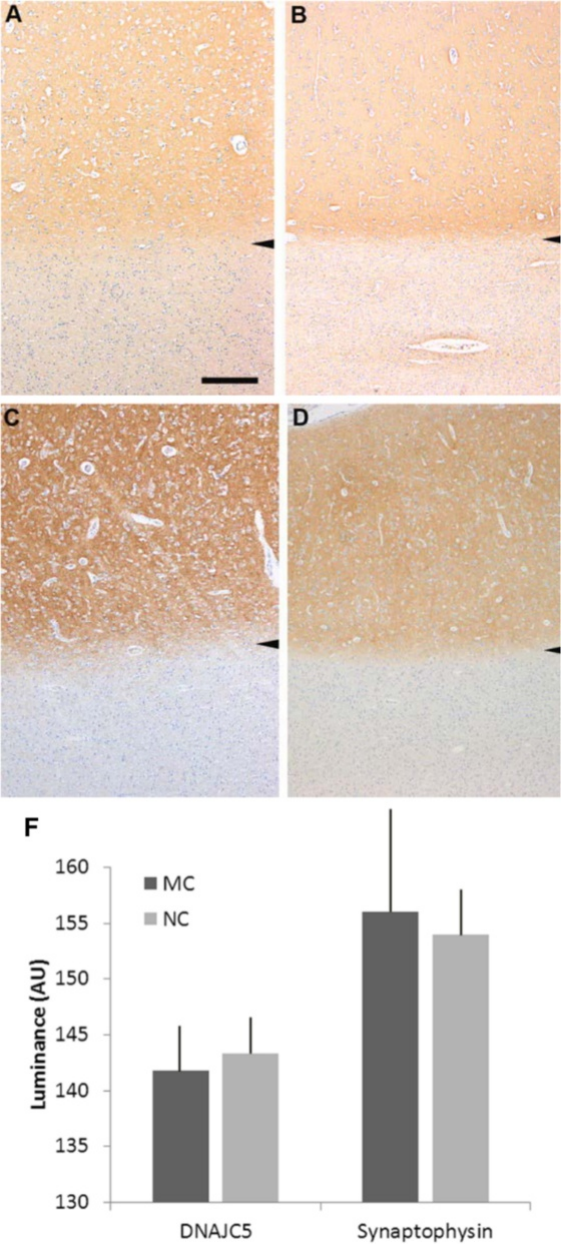
Immunohistochemistry for *CSPα* and synaptophysin in the cerebral cortex of an early stage AD-ANCL patient (**a**) *DNAJC5/CSPα* immunohistochemistry in the cerebral cortex of an early stage AD-ANCL patient (*zone of staining above arrowhead*); the underlying white matter is unstained. **b** non-mutation control subject. **c** Immunohistochemistry for synaptophysin in the cerebral cortex of an early stage AD-ANCL patient (*zone of staining above arrowhead*); the underlying white matter is unstained. **d** non-mutation control subject. Bar: 500 μm. **f** Quantification of the intensity of immunoreactivity staining (luminance measured as arbitrary values) (*graph*). Values represent the mean ± S.E.M. of three independent experiments using Student’s *t* test

*DNAJC5/CSPα* (Fig. 5a, b) and synaptophysin (Fig. 5c, d) immunohistochemistry revealed diffuse neuropil staining of the cerebral cortex, consistent with synaptic localization. Densitometry analysis of the *DNAJC5/CSPα* and synaptophysin staining was compared with 10 aged non-mutation carrier (NC) controls (for demographic data see Table 1). Although there was a trend towards a decrease in synapse (synaptophysin) density in AD-ANCL, it was not statistically significant (p = 0.16, two tailed t-test). Densitometry of *DNAJC5/CSPα* immunohistocemistry was also variable, probably due to fixation artifacts, and no difference (p = 0.8, two tailed t-test) in the density of staining between the mutation carriers and non-carriers was observed (Fig. 5f).

### Changes in levels of synaptic proteins in the brains of terminal AD-ANCL patients

**Fig. 6:**
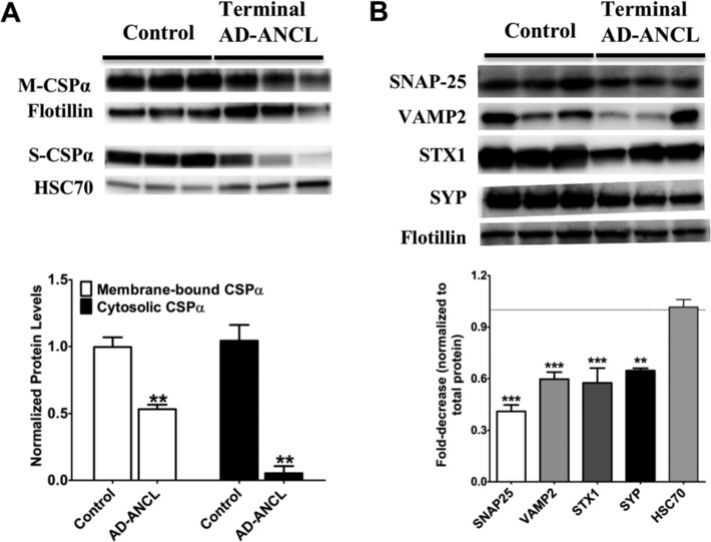
Presynaptic protein levels in the brains of terminal AD-ANCL patients. **a** Representative western blots (*top*) and semi-quantitative analysis (*graph, bottom*) displaying the protein levels of *CSPα* in the membrane (M-CSPα) (normalized to Flotillin) and cytosolic/soluble (S-CSPα) (normalized to *HSC70*) fractions from the occipital lobe of three controls and three terminal AD-ANCL patients. The graphs show the expression level of the indicated proteins normalized to flotillin or *HSC70* expression. **b** Representative western blots (*top*) and semi-quantitative analysis (*graph, bottom*) show the protein levels of SNAP-25, vesicle-associated membrane protein 2 (*VAMP2*/ Synaptobrevin), Syntaxin 1 (*STX1*), and Synaptophysin (*SYP*) in the occipital lobe from three controls and three terminal AD-ANCL patients in the membrane fraction. Values represent the mean ± S.E.M. of three independent experiments. **, *p* ≤ 0.01; ***, *p* ≤ 0.001 using Student’s *t* test

We previously predicted that the p.L115R mutation would dramatically decrease the affinity of *CSPα* for membranes [6]. Here, we show that mutant CSPα-p.L115R reduces levels of *CSPα* in the cytosolic fraction by 95 % (0.05 ± 0.01 S.E., n = 6, p = 0.001, unpaired t-test) compared to controls, while the membrane-bound fraction (detergent soluble) is reduced by ~50 % (0.53 ± 0.03 S.E., n = 6, p = 0.004, unpaired t-test) in the occipital lobe of terminal AD-ANCL patients compared to controls (Fig. 6a). Detailed analysis of CSPα-deficient mice has shown that neurodegeneration in the absence of *CSPα* is a consequence of the defective function of *SNAP-25* and SNARE binding proteins [14]. Therefore, we examined whether the CSPα reduction found in terminal AD-ANCL patients was accompanied by changes in other synaptic proteins. We found a significant reduction in several SNARE-complex forming presynaptic proteins including, but not limited to a 59 % reduction in SNAP-25 levels, 41 % reduction in synaptobrevin/VAMP2 levels and a 43 % reduction in syntaxin 1 levels in brains of terminal AD-ANCL patients compared to controls. We also found a 36 % reduction in synaptophysin levels and no changes in* CSPα*’s partner, *HSC70* levels (Fig. 6a). The reduction of the presynaptic proteins was similar across the different brain regions (frontal, parietal and temporal) or cerebellum among the AD-ANCL patients (Data not shown). No flash frozen tissue was obtained from the early stage AD-ANCL patient, which prevents us from performing the same biochemical analyses.

## Discussion

AD-ANCL is a rare multisystem neurodegenerative disorder characterized by intracellular accumulation of macromolecular debris [4, 5], caused by p.L115R (Fig. 1) and p.L116Δ mutations in *DNAJC5/CLN4B/CSPα* [6, 7, 8]. Nearly all of our understanding of AD-ANCL is based on analysis of postmortem tissue from terminal cases [5, 7]. Here, we describe a 37 year old patient in a clinically early stage of AD-ANCL harboring a p.L115R mutation in *DNAJC5/CSPα.* This patient was a member of a multigenerational family with AD-ANCL, which has been clinically and pathologically described by Josephson et al. [5]. The average age at onset in the other members of this family is 36 ± 2.44 years (range 32–40) with a duration of 9.3 ± 3.3 years (range 5–13). The clinical symptoms start with new-onset generalized tonic–clonic seizures followed by dementia (aprox. 3 years), motor impairment and finally death [5]. Interestingly, most of the histopathological findings found in this patient are indistinguishable from the changes reported in terminal cases, including the clear presence of AFSM in the soma of neurons and most prominent in pyramidal neurons (Fig. 3c) [5]. However, unlike the terminal cases, there was no brain atrophy or significant differences in *CSPα* or synaptophysin in the neuropil compared to the controls (Fig. 5). The effect of these mutations on *CSPα*, a synaptic protein, and how this results in AD-ANCL has not been established. This study demonstrates alterations in the neuronal soma, most likely in the lysosome, of an early-stage of the disease (Fig. 3a-d). In contrast, massive synaptic degeneration was only observed in post-mortem tissue from terminal AD-ANCL patients (Fig. 6a, b) [5, 7], suggesting that the dysfunction in the soma of neurons likely at the lysosomal level occurs before the massive synaptic degeneration as a consequence of the aggregate-derived toxic effects of the mutation in *DNAJC5/CSPα* [16].

Neuronal cell death seems to be correlated with age of onset and duration of the disease in most NCLs [19]. In AD-ANCL there is a remarkable neuronal depletion in the cortex of the frontal, parietal and temporal lobes of the AD-ANCL terminal cases [5]. Here, we found minimal to no evidence of neuronal loss or cortex atrophy in a clinically early-stage patient with AD-ANCL in the examined areas.

Microgliosis and astrogliosis seems to be an early event, possibly even occurring before neuronal degeneration in several NLCs [19, 20] including adult forms [5], and remains remarkable in very advanced stages of neurodegeneration in the cerebral cortex [5, 19]. Here, we found microglial and astroglial activation in isolated areas surrounding the swollen neurons in the cortex of an early-stage AD-ANCL patient.

Here, we report that terminal AD-ANCL patients exhibit reductions in presynaptic proteins that form the SNARE complex including *SNAP-25*, *VAMP2* and *STX1* (Fig. 6a, b). A similar reduction in *SNAP-25* was reported in CSPα-deficient mice [9, 10, 14] but unlike the findings in CSPα-deficient mice, terminal AD-ANCL patients exhibit similar HSC70 levels compared to control (Fig. 5b). Other LSDs also exhibit SNARE defects as a consequence of the primary lysosomal defects [21]. Unfortunately, lysosome function has not been evaluated in CSPα-deficient mice [12, 16]. In contrast, CSPα-p.L115R induces dramatic changes in the soma that appear to precede the synaptic defects. This sequence of events has been reported in other LSDs where non-lysosomal mutant proteins affect the intracellular trafficking of proteins upstream or downstream of the lysosome, including synaptic proteins, subsequently producing synaptic dysfunction [22–24].

We have confirmed and extended the finding that postmortem brain samples from terminal AD-ANCL patients display a large reduction in *CSPα* levels compared to controls (Fig. 6a) [7, 17, 25]. Interestingly, the levels of membrane-bound *CSPα* are decreased 50 % in terminal AD-ANCL patients (Fig. 6a). CSPα hemizygous mice exhibit a 50 % reduction in the level of CSPα and are phenotypically normal [10]. Assuming membrane-bound *CSPα* is the main functional fraction, this 50 % reduction alone would not explain the synaptic changes and neurodegeneration found in terminal AD-ANCL patients (Fig. 6a, b). This suggests that an additional mechanism other than an isolated reduction in *CSPα* levels is responsible for the neurodegeneration found in AD-ANCL. However, we cannot rule out the possibility that the overall reduction, up to one third of the control levels, in both soluble and membrane-bound *CSPα* found in AD-ANCL patients could be responsible for the synaptic defects seen.

We also found in brain homogenates from individual controls that there is a substantial amount of *CSPα* present in the soluble/cytosolic fraction (Fig. 6a). *CSPα* levels in this particular fraction are dramatically reduced in AD-ANCL patients (Fig. 6a). The cytosolic *CSPα* fraction extracts from the rat brain have been described to interact with *HSC70* [26]. The amount of soluble/cytosolic *CSPα* depends on both the cell type [27–29] and the activity of palmitoyl- transferases on CSPα [30]. Thus, it is possible that human brain expression levels and types of palmitoyl- transferases vary in different cell types [31] or that palmitoyl- transferase activity is age-dependent resulting in a greater soluble/cytosolic *CSPα* fraction. We cannot rule out the possibility that this finding is an artifact or contamination from small vesicles in our detergent-free fraction. However, the CSPα levels found in three different individual controls strongly suggest that this is not an artifact (Fig. 6a). The role of soluble/cytosolic *CSPα* is not clearly defined since the most thoroughly studied is membrane-bound *CSPα*. However, *CSPα* exhibits both chaperone and co-chaperone activity that does not depend on its ability to bind the membrane.

The understanding of how mutations in a “synaptic” protein can lead to lysosome dysfunction is currently unclear. Lysosomes are dynamic organelles that receive and degrade macromolecules from secretory, endocytic, autophagic and phagocytic membrane-trafficking pathways [32]. Lysosomes also serve as a common endpoint for multiple vesicle-based trafficking systems [32]. CSPα is critical for maintaining vesicle-membrane fusion events [28]. The fusion events between lysosomes and endosomes, phagosomes, autophagosomes or plasma membrane, are all mediated by the SNARE complexes [32]. The main proteins forming these *trans*-SNARE complexes include syntaxin-7, syntaxin-8 (Q-SNARES) and vesicle-associated membrane protein-8 (*VAMP8*) and *VAMP7* (R-SNARES), in addition to synaptosome-associated protein of 23 kDa (*SNAP23*), synaptotagmin-VII and *Rab3a* [32], some of which have been identified as *CSPα*’s partners [16]. Terminal AD-ANCL patients exhibit significant reductions in SNARE-complex forming proteins (Fig. 6b). Thus, it is likely that dysfunctional CSPα affects the machinery that coordinates these lysosomal fusion events resulting in the formation of the ceroid aggregates and subsequent swelling of the cellular soma (cortical pyramidal neurons and palmar sweat gland) seen in AD-ANCL.

In this report, we describe the clinical and pathological features of a patient in a clinically early stage of AD-ANCL with all the pathological cellular changes found in terminal AD-ANCL cases, but with minimal or no apparent reduction in *CSPα* or synaptophysin in the neuropil. In contrast, terminal AD-ANCL patients exhibit significant reductions of presynaptic protein levels, including a significant reduction in *CSPα* and its main partner *SNAP-25* but not in *HSC70* levels. Our analysis of a single brain region in an early-stage AD-ANCL patient showed a non-significant trend toward synaptic loss (Fig. 5a). *CSPα*-deficient mice exhibit a massive neurodegeneration with wide heterogeneity in the time-course and type of neurons affected [10]. Thus, it seems possible that this early-stage AD-ANCL patient might have synaptic losses that are not obvious in gross immunohistological analyses.

In summary, the widespread neurodegeneration seen in terminal AD-ANCL patients seems to be a consequence of both the early effects of mutant *CSPα* in the soma, most likely on lysosome function, and subsequent synaptic dysfunction resulting from the reduction in *CSPα* levels. We hypothesize that in this AD-ANCL patient the lysosomal dysfunction (ceroid accumulation) occurred before neuronal cell death and synaptic degeneration.
